# Emotion suppression differentially moderates the link between stress and cardiovascular disease risk in Japanese and Americans

**DOI:** 10.1016/j.ijchp.2025.100555

**Published:** 2025-02-27

**Authors:** Darcianne K. Watanabe, Shinobu Kitayama, DeWayne P. Williams, Julian F. Thayer

**Affiliations:** a School of Social Ecology, c/o Department of Psychological Science, University of California, Irvine, 4201 Social and Behavioral Sciences Gateway, Irvine, CA, 92697, USA; b Department of Psychology, University of Michigan, 1004 East Hall, 530 Church Street, Ann Arbor, MI, 48109, USA; c Department of Psychological Science, University of California, Irvine, 4201 Social and Behavioral Sciences Gateway, Irvine, CA, 92697, USA

**Keywords:** Adaptive suppression, Cultural differences, Stress and cardiovascular health, Pro-inflammatory cytokines, Interleukin-6, C-reactive protein, MIDUS, MIDJA 2, Emotion regulation, Reappraisal

## Abstract

**Background:**

Cardiovascular disease (CVD) remains a key cause of mortality worldwide. Prior work has found that the association between stress and cardiovascular outcomes is moderated by emotion regulation (ER) and expressive suppression (i.e., emotion inhibition), which is linked with adverse outcomes (i.e., inflammation) in Western (Americans) but not Eastern (Japanese) populations. Existing cultural differences in biological stress responses and suppression use suggest that these factors may have different implications for CV outcomes.

**Objective:**

We address this gap in the literature by examining if ER differentially moderates the relationship between stress and CVD risk between Japanese and American adults.

**Method:**

Participants were from the Midlife in Japan and Midlife in the United States studies and had complete biomarker and psychological data (Japanese: *N* = 315, *M*_age_ = 59.22, 149 females; Americans: *N* = 524, *M*_age_ = 51.98, 291 females). Stress was indexed using the perceived stress scale. Trait suppression and reappraisal were indexed using the Emotion Regulation Questionnaire. CVD risk was indexed using a composite score of body mass index, C-reactive protein, interleukin-6, systolic blood pressure, and high-density lipoprotein cholesterol ratio.

**Results:**

Adjusting for age, sex, education, tobacco, alcohol, and prescription medication use, linear regressions revealed robust cultural differences among those with high suppression (*r =* -0.10 [-0.19, -0.01]). Higher stress was linked with higher CVD risk in Americans regardless of the level of reappraisal or suppression (r's > 0.11, p's < 0.07). In contrast, among Japanese with high suppression, higher stress was associated with lower CVD risk (*r=* -0.09 [-0.23, 0.05]). Higher stress was associated with greater inflammation among Japanese with lower suppression (*r=* 0.10 [-0.07, 0.28]).

**Conclusions:**

Consistent with prior work, these findings suggest that adaptive ER moderates the association between stress and CVD risk, and that suppression may not be universally ‘maladaptive.’ Results emphasize the importance of considering cultural context when assessing the impact of emotion suppression on health, which may help explain differences in CVD outcomes between individuals from Eastern and Western populations.

## *Introduction*

Cardiovascular disease (CVD) is the primary cause of mortality in the United States and the second in Japan (Ahmad et al., 2023; Global Health Data Exchange (GHDx), 2023; Ohira, Eguchi, Hayashi, Kinuta, & Imano, 2024). Previous research has suggested that the association between stress and cardiovascular outcomes is moderated by emotion regulation, defined as one's ability to manage, express, and experience emotions in response to contextual demands (Cole et al., 1994; Gross, 1998; Roy et al., 2018). However, there are crucial cultural differences between Japanese and Americans in biological stress responses and how emotions are regulated, which may have different implications for cardiovascular outcomes (Coe et al., 2011; Markus & Kitayama, 1991; Matsumoto, Yoo, Fontaine et al., 2008; Miyamoto et al., 2013; Park et al., 2020). Thus, to address the gap in the literature, we examined cross-cultural differences in the moderating role of emotion regulation in the relationship between stress and CVD risk.

Stressful life experiences have been linked with chronic systemic or arterial inflammation, which are putative causes of atherosclerotic CVD in developed countries, including the U.S. and Japan (Abboud & Singh, 2017; Abboud et al., 2012; Hansson, 2005; Singh et al., 2014). Although Japanese have a lower estimated lifetime risk of atherosclerotic CVD (i.e., coronary heart disease) (Turin et al., 2016), elevated risk of CVD has been linked with modifiable risk factors common in Western (i.e., American) and Eastern (i.e., Japanese) populations including diabetes mellitus, dyslipidemia, and hypertension (Fox et al., 2008; Imai et al., 2021; Peng et al., 2023; Turin et al., 2016). Thus, adiposity, blood pressure (BP), pro-inflammatory markers, and serum lipid levels are common biomarkers used in Japanese and American studies on CVD risk (Lu et al., 2014; Nakamura et al., 2001). The American College of Cardiology, American Heart Association, and Japan Atherosclerosis Society suggest that the prevention of CVD risk may be achieved by reducing inflammation via lowering blood pressure, total cholesterol, and obesity, and increasing physical inactivity (Arnett et al., 2019; Okamura et al., 2023), and clinical interventions to modify behavioral tendencies, such as emotion regulation strategies, may also improve health outcomes (Roberts et al., 2017). However, to minimize health risks in Eastern and Western populations, a more comprehensive understanding of cross-cultural differences in the relationships between stress, emotion regulation, and CVD risk is needed.

Perceived stress, defined as the perception of an inability to cope with environmental demands (i.e., stressors) (Cohen et al., 1983; Lazarus, 1966; Lazarus, 1974; Lazarus & Folkman, 1984), is one factor linked with CVD risk via inflammation and other mechanisms (Dar et al., 2019; Jurgens et al., 2023; Rosengren et al., 2004; Roy et al., 2018; Sara et al., 2022). Western individuals exposed to chronic stress show elevated levels of pro-inflammatory cytokines, interleukin-6 [IL-6], and C-reactive protein [CRP] (Jurgens et al., 2023; Marsland et al., 2017), whereas healthier inflammatory profiles have been found among Eastern individuals (Coe et al., 2011) signaling lower CVD risk. These findings indicate that stress responses (i.e., inflammation) may differ cross-culturally and that cultural factors (i.e., shared values, beliefs, and practices) may moderate the link between stress and CVD risk.

Emotion regulation, which varies by culture, is one factor that may mitigate the effects of stress on CVD risk (Appleton & Kubzansky, 2014; Ekman, 1972; Hofstede, 1980; Markus & Kitayama, 2010; Matsumoto, Yoo, Nakagawa et al., 2008; Mesquita & Frijda, 1992). Emotion regulation strategies are developed through socialization, including shared cultural values and practices (Gross & John, 2003; John & Gross, 2004; Kitayama et al., 1997; Markus & Kitayama, 1991; Matsumoto, Yoo, Nakagawa et al., 2008). In this cultural context, values common in Japanese and American cultures, such as interdependence and independence, respectively, may then motivate and influence how individuals regulate their emotions (H. S. Kim & Sherman, 2007; Kitayama et al., 1997; Markus & Kitayama, 1991; Oyserman et al., 2002). For example, expressive suppression (i.e., inhibition of emotions), a regulatory strategy that Eastern individuals use more frequently, may facilitate interconnection. In contrast, cognitive reappraisal (i.e., emotional reframing) is more commonly used in Western cultures as it may lead to the expression of more positive emotions, which are perceived as desirable (Chen et al., 2020; Matsumoto, 1990, 2008; Ramzan & Amjad, 2017; Soto et al., 2011; Sun & Lau, 2018; Tsai & Lu, 2018; Weiss et al., 2022). Notably, whereas reappraisal is generally regarded as ‘adaptive,’ suppression is often characterized as ‘maladaptive’ (Aldao et al., 2010; Gross & John, 2003; Troy et al., 2018). In Americans, suppression has been positively linked with elevated physiological responses (Butler et al., 2003; Gross & Ford, 2023; Gross & John, 2003) to laboratory stress (Tyra et al., 2023) and meta-analytically to daily-life negative emotions (Boemo et al., 2022), both of which have been linked with adverse cardiovascular outcomes and markers of CVD (Appleton & Kubzansky, 2014; Appleton et al., 2013; Nabi et al., 2008; Ospina et al., 2022). However, examinations of negative affect, which is positively associated with suppression (Gross & John, 2003), showed that it was associated with elevated markers of CVD risk (i.e., IL-6, CRP, systolic blood pressure [SBP], and the ratio of total to high-density lipoprotein cholesterol [T/HDL]) among Americans but not Japanese (Curhan et al., 2014; Kitayama et al., 2015; Miyamoto et al., 2013; Park et al., 2020). It is important to note that while the process of emotion regulation involves both the experience of emotions (Watson et al., 1988) and how individuals manage their emotions (Cole et al., 1994; Gross, 1998), an examination of the latter may be particularly critical for cardiovascular outcomes (Appleton & Kubzansky, 2014; Appleton et al., 2013; Haukkala et al., 2010).

To our knowledge, only one study has examined the role of emotion regulation in the link between stress and CVD risk, but cultural differences were not investigated. Roy et al. (2018) found that perceived stress, as measured by the Perceived Stress Scale (Cohen et al., 1983) was differentially associated with CVD risk, indexed by a composite CVD risk score adapted from the AHA's ideal health score calculation (Lloyd-Jones et al., 2010), as a function of emotion regulation difficulties, as measured by the Difficulties in Emotion Regulation Scale (Gratz & Roemer, 2004) Americans with low emotion regulation difficulties had relatively lower CVD risk (*ß* = 0.007, *r* = 0.06 [−0.14, 0.25]) than those with high emotion regulation difficulties (*ß* = 0.032, *p =* *0*.014, *r* = 0.08 [−0.11, 0.27]) (Roy et al., 2018). These findings suggest that the manner in which individuals regulate their emotions may influence the impact stress has on cardiovascular outcomes. However, whether findings by Roy and Colleagues (2018) generalize beyond Western populations has not been examined. Taken together, if cultural differences exist in biomarkers of stress (e.g., inflammation and cortisol) and CVD prevalence, and the relationship between markers of CVD risk and negative affect, which is linked with particular emotion regulation strategies (i.e., suppression) differs by culture, it seems plausible that there may also be cultural differences in the relationships between stress, emotion regulation (e.g., suppression), and CVD risk (Boemo et al., 2022; Curhan et al., 2014; Gross & John, 2003; Kitayama et al., 2015; Miyamoto et al., 2013; Park et al., 2020; Turin et al., 2016).

The integration of the Neurovisceral Integration Model and the Neuro-Culture Interaction Model provides a comprehensive framework for understanding how cultural differences in emotion regulation may contribute to differential health outcomes (Kitayama & Uskul, 2011; Smith et al., 2017; Thayer & Lane, 2000; Thayer et al., 2021). The Neurovisceral Integration Model explains how the central autonomic network, particularly the prefrontal-amygdala circuit, coordinates adaptive affective, physiological, and psychological processes (Smith et al., 2017; Thayer & Lane, 2000). When ‘maladaptive’ emotion regulation strategies, such as suppression, are employed in response to stress, Americans typically show increased amygdala activity and decreased frontal cortex activity, and defensive physiological responses such as elevated blood pressure, heart rate, cortisol levels, and pro-inflammatory markers, all of which contribute to CVD risk (Appleton et al., 2013; Brosschot et al., 2015; Butler et al., 2009; Era et al., 2021; Gianaros & Sheu, 2009; Gianaros et al., 2008; Grupe & Nitschke, 2013; Kivimäki & Steptoe, 2018; Marsland et al., 2017; McDade et al., 2006; Saha et al., 2000; Tawakol et al., 2017; Taylor et al., 2008; Urry et al., 2006). Complementing the Neurovisceral Integration Model, the Neuro-Culture Interaction Model posits that cultural practices, like the habitual use of suppression to maintain social harmony, reshape neurobiological pathways and brain connectivity (Chiao et al., 2013; Kitayama & Park, 2010; Kitayama & Uskul, 2011; Schwartz & Begley, 2002). These cultural practices are evident in parental socialization, which may shape the emotion regulation strategies encouraged in early childhood (Kim & Sherman, 2007; Kitayama & Uskul, 2011; Kitayama et al., 1997; Lebra, 1976; Markus & Kitayama, 2010; Matsumoto, 1993; Rochanavibhata & Marian, 2023; Salih et al., 2023; Zahn-Waxler et al., 1996). Given the close synchrony between parents and children, parental influences may extend beyond behavior to shape children's neurobiological mechanisms of emotion regulation and stress responses (Gee et al., 2014; Reindl et al., 2018). Indeed, cross-cultural neuroimaging studies further suggest that adults from Eastern and Western cultures exhibit distinct physiological responses to stress and emotion regulation, potentially reflecting culturally specific socialization practices (Chiao et al., 2013; Varnum, Shi, Chen, Qiu, & Han, 2014). Consequently, as cultural practices shape emotion regulation strategies, neural processes and pathways may also differ as a function of cultural norms (Chiao et al., 2013; Kitayama & Park, 2010; Kitayama & Uskul, 2011; Schwartz & Begley, 2002; Varnum, Shi, Chen, Qiu, & Han, 2014). Thus, it is reasonable to anticipate that culture influences the interaction between stress and emotion regulation, leading to differential physiological responses. When considered together, the Neurovisceral Integration Model and Neuro-Culture Interaction Model models provide a framework for understanding how cultural practices shape neural pathways related to emotion regulation and stress, ultimately contributing to cross-cultural differences in physiological responses (Kitayama & Uskul, 2011; Smith et al., 2017; Thayer & Lane, 2000; Thayer et al., 2021). Thus, understanding how emotion regulation and stress responses are shaped by culture may illuminate the sociocultural mechanisms underlying differences in CVD risk among Eastern and Western populations.

### Present study

The present study builds on prior work showing 1) cultural differences in the link between CVD risk factors and negative affect (Miyamoto et al., 2013; Park et al., 2020) and 2) evidence in Americans linking higher perceived stress and emotion regulation difficulties with higher CVD risk (Roy et al., 2018). We examined the magnitude of difference between Japanese and Americans in the association between stress and two emotion regulation strategies correlated with emotion regulation difficulties and negative affect, expressive suppression and cognitive reappraisal, on CVD risk (Boemo et al., 2022; Gross & John, 2003; Sörman et al., 2022; Zelkowitz & Cole, 2016). To further extend the work of Miyamoto et al. (2013) and Park et al. (2020), our composite index of CVD risk included five biomarkers. Based on prior reports, we hypothesized that Japanese and Americans would differ in their use of suppression, which would moderate the association between stress and CVD risk differentially as a function of culture (Kitayama & Uskul, 2011; Roy et al., 2018; Smith et al., 2017; Thayer & Lane, 2000). Since elevated inflammation has been linked with stress (Jurgens), emotion regulation (Appleton et al., 2013; Ellis et al., 2019), and CVD risk (Kaptoge et al., 2014; Li et al., 2017; Marsland et al., 2017), our secondary aim examined CVD risk indexed by inflammatory biomarkers, IL-6 and CRP (see Supplemental Material). No hypotheses were generated for the exploratory analyses with inflammation. We focused on interpreting effect sizes and confidence intervals as Amrhein et al. (2019) and Lipsey et al. (2012) recommended.

## *Materials and methods*

### Participants and procedures

American and Japanese participants’ data were obtained from the MIDUS Refresher Survey and Biomarker projects (*n* = 863), whereas the Japanese data were obtained from the MIDJA, wave 2 (*n* = 657; MIDJA 2). The Institutional Review Boards approved data collection at MIDUS study sites (University of California, Los Angeles [UCLA]; University of Wisconsin, Madison [UW]; and Georgetown University [GU]), and informed consent was obtained from all participants. Recruitment, enrollment, consent, and procedures related to the MIDUS and MIDJA are only briefly outlined below as they are described in detail elsewhere (Dienberg Love et al., 2010; Kitayama, Karasawa et al., 2010; Ospina et al., 2022; Park et al., 2023).

The MIDUS Refresher survey and biomarker data were collected from 2011 to 2016. Survey data collection included a phone interview (30 mins) and two Self-Administered Questionnaires (SAQ). The biomarker data collection occurred during an overnight stay at a MIDUS regional site based on where the participant resided (UCLA, UW, or GU). Medical history, physical exam with vital signs, and SAQ were completed on Day 1, whereas Day 2 included a fasting blood draw and functional assessment, among other assessments. Informed consent was obtained via phone and in writing at the clinic visit.

MIDJA 2 was conducted from 2012 to 2014 in two phases. The first phase included an SAQ with several measures (e.g., perceived stress and history of tobacco use), which was administered in 2012. The second phase was conducted from 2013 to 2014 and included biomarker assessments. Biomarker data collection (blood and urine) occurred at local clinics (45 to 60-minute sessions) and during at-home biomarker assessments (saliva). During the at-home session, medical history and additional psychosocial evaluations were obtained via a second SAQ. To achieve equivalent meaning in Japanese and English, all scales were first translated into Japanese and then back-translated to English (Kitayama, Karasawa et al., 2010). The Japanese version was used in Japan. MIDJA 2 participants were compensated 3000 yen (∼$28–30) for completing the surveys and 10,000 yen (∼$30) after clinic assessments. We chose to focus on MIDJA 2 participants to account for similar participant experiences related to world events (e.g., economic recession), as MIDJA 2 data were collected in close temporal proximity to the MIDUS Refresher data.

### Perceived stress

Perceptions about stressful situations in individuals’ lives over the prior month were measured using the 10-item Perceived Stress Scale (PSS; “In the last month, how often have you found that you could not cope with all the things that you had to do?”) collected as part of the survey phase (Cohen & Williamson, 1988; Cohen et al., 1983). Responses are on a 5-point scale ranging from “never” (1 point) to “very often” (5 points). Four positively phrased items are reverse scored. Total scores range from 0 to 40, with lower scores indicating lesser perceived stress. The PSS showed excellent internal consistency in the current investigation (MIDJA α = 0.82; MIDUS α = 0.87), and its construct validity has been demonstrated in Japanese and American populations (Lee, 2012; Mimura & Griffiths, 2004).

### Emotion regulation

Emotion regulation was measured using a shortened version of the Emotion Regulation Questionnaire (ERQ; Gross & John, 2003) administered prior to the MIDUS and MIDJA biomarker assessments (Finley et al., 2024). ERQ reappraisal items: “I control my emotions by changing the way I think about the situation I'm in” and “When I'm faced with a stressful situation, I make myself think about it in a way that helps me stay calm.” and ERQ suppression items: “When I am feeling negative emotions (such as sadness or anger), I make sure not to express them” and “I keep my emotions to myself.” Suppression showed acceptable internal consistency (MIDJA α = 0.69; MIDUS α = 0.73), as did reappraisal (MIDJA α = 0.77; and MIDUS α = 0.54).

### Cardiovascular disease risk

Following prior work (Kitayama et al., 2015, 2018; Park et al., 2020; Roy et al., 2018) five biomarkers were used to comprise a CVD risk score, including: BMI (weight [in kilograms] by height [in meters] squared, CRP (mg/L), IL-6 (mg/L), SBP (mm/Hg), and T/HDL (mg/dL). T/HDL was used instead of the ratio of low-density lipoprotein cholesterol (LDL-C) to HDL (LDL/HDL) as 1) estimates of LDL-C are less accurate (Martin et al., 2013; Schectman & Sasse 1993); 2) T/HDL has been found to better predict heart disease risk than LDL/HDL (Lamarche et al., 1996; Lemieux et al., 2001); and 3) T/HDL and is more commonly used in clinical practice. Biomarker assessments occurred approximately one year after the SAQ. These procedures are briefly described below as they are described in detail elsewhere (Kitayama et al., 2015; Park et al., 2020, 2023).

To maintain consistency with the MIDUS biomarker protocol, Japanese blood samples were prepared as frozen aliquots and sent to three U.S. laboratories. Cholesterol (Total and HDL) and CRP assays were performed at Meriter Laboratories (Madison, WI) and the Laboratory for Clinical Biochemistry Research (University of Vermont, Burlington, VT), respectively, and IL-6 was assayed at the MIDUS Biocore Laboratory (University of Wisconsin, Madison, WI). Serum total cholesterol (minimum 3.86 mg/dL) and HDL-cholesterol (minimum 3 mg/dL) were analyzed using a Cobas Integra® analyzer (Roche Diagnostics, Indianapolis, IN). Plasma CRP was initially assayed using the Dade Behring (Schwalbach, Germany). If they fell below the CRP assay range (0.014–216 ug/mL, min 10–6 ug/mL), they were re-assayed using a high-sensitivity kit (Meso Scale Diagnostics, Rockville, MD) with a lower sensitivity of detection at 0.00024 ug/L. Serum IL-6 levels were determined using a high-sensitivity enzyme-linked immunosorbent assay kit (ELISA) with a minimum sensitivity of detection at 0.156 pg/mL (Quantikine, R&D Systems, Minneapolis, MN). A Cobas Integra analyzer (Roche Diagnostics, Indianapolis, IN) was used to assay cholesterol data (total and HDL). Reference ranges were <200 and >40 mg/dL for total and HDL, respectively. Height, weight, and blood pressure were collected during the clinic visit. Three measurements of resting and seated BP were taken with a 30-second pause between five-minute readings. The second and third blood pressure measurements were used to calculate the average SBP.

Our composite CVD risk score was adapted from the American Heart Association's ideal health score calculation (Lloyd-Jones et al., 2010; Roy et al., 2018). Separate scores were calculated for Americans and Japanese. For each risk factor, zero points were assigned for low risk (BMI: ≤ 24 kg/m2; CRP: <1 mg/L, and SBP: <120 mmHg), one point for moderate risk (BMI 25–30 kg/m2; CRP: 1 mg/*L* > 3 mg/L; SBP: 120–140 mmHg) and two points for high risk (BMI >30 kg/m2; CRP: > 3 mg/L; and SBP ≥ 140 mmHg). Risk strata for IL-6 and T/HDL were computed using separate tertiles for each cultural group. Points were assigned as previously described. Japanese risk strata were defined as low: T/HDL <2.74 mg/dL, IL-6 < 0.82 pg/mL; moderate: T/HDL 2.74–3.57 mg/dL, IL-6 0.82–1.42 pg/mL; and high: T/HDL >3.57 mg/dL, IL-6 > 1.42 pg/mL. Americans risk strata were defined as low: T/HDL <2.60 mg/dL, IL-6 < 1.3 pg/mL; moderate: T/HDL 2.60–3.48 mg/dL, IL-6 1.3–2.7 pg/mL; and high: T/HDL >3.48 mg/dL, IL-6 > 2.7 pg/mL.

### Covariates

Relevant covariates of inflammatory markers and elevated cardiovascular risk included age, sex, current smoker (yes/no), frequency of alcohol use, educational attainment, and prescription medication use (yes/no) (Coe et al., 2011; O'Connor & Irwin, 2010; O'Connor et al., 2009). Weekly alcohol consumption was coded as every day (one point) to less than one day a week (five points). Following prior work, educational attainment was rescaled to a 7-point scale (1 = 8th grade, junior high school, 7 = attended or graduated from graduate school) to make the scores comparable across cultural groups (Kitayama et al., 2015; Park et al., 2020, 2023).

### Data analysis

Japanese and American participants with complete data on all variables of interest were included in our analyses (*n* = 839). To examine cultural differences between our variables of interest, we conducted independent means *t*-tests by cultural group. Analyzed sample sizes, means, t-values, p-values (two-tailed alpha = 0.05), effect sizes, and 95% CIs are reported in Table 1. In all analyses, Fisher's r to z transformation was used to assess the magnitude of difference between the associations for each cultural group (Lenhard & Lenhard, 2022; Lowry, 2001; Meng et al., 1992), effect sizes (r), and 95% confidence intervals (in brackets) were obtained using the Psychometrica effect size calculator (P. D. Ellis, 2010; Rosenthal & DiMatteo, 2001) Pearson's zero-order correlations were computed separately for each cultural group. Partial correlations, controlling for covariates age, sex, smoking status, alcohol consumption, educational attainment, and prescription medication use, were also examined.

**Table 1:** Descriptive statistics stratified by culture.

	**Japanese (*n* = 315)**		**Americans (*n* = 524)**					
	M	SD	M	SD	*t*	*r*	95% CI	*p*
Age	59.22	13.01	51.98	13.58	7.60	.25	0.19, 0.32	**<0.001**
Smoke	1.86	0.35	1.90	0.31	−1.51	.05	−0.02, 0.12	.13
Alcohol	3.83	2.00	3.69	1.29	1.28	.04	−0.02, 0.11	.20
Education	4.21	1.62	5.55	1.47	−12.28	.39	0.32, 0.46	**<0.001**
Rx	0.59	0.49	0.72	0.45	−3.85	.13	0.06, 0.20	**<0.001**
Sex (Women/Men)	149/166		291/233		*X2 (839) =* 5.35			**.02**
Perceived Stress	25.88	5.92	21.98	6.17	8.99	.30	0.23, 0.36	**<0.001**
Reappraisal	10.13	1.80	9.98	2.16	1.04	.04	−0.03, 0.10	.30
Suppression	9.05	2.13	7.78	2.75	7.04	.24	0.17, 0.30	**<0.001**
CV Risk	3.62	2.20	4.89	2.51	−7.43	.25	0.18, 0.32	**<0.001**
IL6 (pg/mL)	0.11	0.78	0.63	0.80	−9.29	.31	0.24, 0.37	**<0.001**
CRP (mg/L)	−0.89	1.18	0.20	1.20	−12.85	.41	0.34, 0.47	**<0.001**
SBP (mm/Hg)	127.91	16.70	126.94	15.73	0.85	.03	−0.04, 0.10	.40
T/HDL (mg/dL)	3.39	1.18	3.25	1.11	2.10	.07	0.00, 0.14	**.04**
BMI (kg/m2)	22.98	3.14	29.27	6.74	−15.58	.47	0.41, 0.54	**<0.001**

Note: Table 1 provides Pearson Chi-Square (*X*^2^) for differences between women and men. Means (M), standard deviations (SD), t-values (t), effect size (r), associated 95% confidence intervals for r's (CI), and p-values (p) are provided for age in years, smoking status (smoke; yes [1], no [2]), frequency of alcohol consumption (alcohol; reverse coded: 1 = every day; 5 = less than 1 day per week), educational attainment (education; (1 = 8th grade, junior high school, 7 = attended or graduated from graduate school), prescription medication use (Rx; yes [1]/no [0]), stress (Perceived Stress Scale; Cohen et al., 1988); cognitive reappraisal and expressive suppression measured using a shortened version of the Emotion Regulation Questionnaire (Gross & John, 2003); natural log-transformed interleukin-6 in pg/mL (IL-6); natural log-transformed C-reactive protein in mg/L (CRP); systolic blood pressure in mm/Hg (SBP); ratio of total to high-density lipoprotein cholesterol in mg/dL (T/HDL); body mass index in kg/m2 (BMI); and composite CVD risk score (CVD Risk).

Prior work conducted moderation analyses with median-split moderators (i.e., emotion regulation; Roy et al., 2018). Despite critiques of median split analyses (e.g., Cohen, 1983), recent work using Monte Carlo studies showed that while median splits sometimes produce more conservative results, the impact on the coefficients is very small. Importantly, in the absence of multicollinearity, no bias towards type I error occurs, whereas in the presence of multicollinearity, the potential deleterious effects are very small (Iacobucci et al., 2014, 2015). For instance, in ANOVA, which is often used for orthogonal factors, median splits do not increase the likelihood of spurious main effects or interaction effects. Thus, we conducted multicollinearity diagnostics. Results indicated tolerance and variance inflation factors for all coefficients in both the Japanese and American samples of <3.8, which is below the multicollinearity threshold of 5 to 10 (Kim, 2019; Menard, 1995). Thus, median scores were computed separately for Japanese and Americans. Participants were categorized as having low scores if their scores fell below the median and having high scores if they included and exceeded the median. We then examined the interactions between PSS and the median-split moderators on our composite CVD risk score in separate linear regression analyses for each cultural group, following prior work (Roy et al., 2018). Simple slope tests were performed (Lowry, 2001; Meng et al., 1992) and test statistics are reported. Sensitivity analyses were conducted to determine whether excluding the covariate prescription medications, including those that might influence our dependent variables, would change our interpretations.

All analyses were conducted using the Statistical Package of Social Sciences (SPSS ver. 28, IBM, Chicago, IL, USA). SPSS PROCESS Model 1 was used for the moderation analyses (IBM SPSS Statistics for Macintosh, 2021). Natural log-transformed IL-6, CRP, and T/HDL measures were used to fit assumptions of linear analyses. We evaluated extreme scores using a global index of influence, the standardized difference in fit (DFFITS) (Belsley, Kuh, & Welsch, 1980), and none of the participants were exerting an undue influence on the overall model fit. All tests used a *p =* *0*.05 level of statistical significance.

## *Results*

### Demographic characteristics

Descriptive statistics for Japanese and Americans are presented in Table 1. Our total sample (*n* = 839) included 315 Japanese (*M*_age_ = 59.22 [13.01], 149 females)] and 524 Americans (*M*_age_ = 51.98 [13.58], 291 females). There were significantly more Japanese men and American women (*X*^2^ [839] = 5.35, *p =* .02). Men had significantly higher CVD risk scores, IL-6, T/HDL, SBP, BMI, and were younger relative to women (each *r >* 0.13, *p <* .03). Women reported significantly lower suppression (each *r* = 0.10, *p <* *0*.004) and higher stress (*r* = 0.11, *p =* .002] than men. Japanese were around three times as likely to have low BMI compared to Americans, and Americans were around four times as likely to have high BMI compared to Japanese (*X*^2^ [839] = 185.66, *p <* .001).

Japanese reported significantly higher stress (*r* = 0.30 [0.23, 0.36]), and Americans had significantly higher CVD risk scores (*r* = 0.25 [0.18, 0.32]). Japanese reported significantly greater suppression use than Americans (*r* = 0.24 [0.17, 0.30]), and this effect size was more than double that found in prior work (Chen et al., 2020). However, no significant differences were found on reappraisal (*r* = 0.04 [−0.03, 0.12]). Japanese had significantly higher T/HDL and significantly lower BMI, CRP, and IL-6 and were older relative to Americans (each *r >* 0.07).

### Cultural differences in reappraisal and suppression

The unadjusted inverse stress-reappraisal association was significant in both cultural groups (each *r >* −0.19, *p <* .001; Table 2). In Japanese, the significant negative unadjusted stress-suppression association (*r =* −0.25 [−0.11, −0.09], *p =* .03) was attenuated after adjustment (*r =* −0.08 [−0.34, −0.14], *p =* .14; Table 3). In Americans, the unadjusted positive stress-suppression association (*r* = 0.07 [−0.01, 0.16], *p =* .09) remained marginally significant after adjustment (*r* = 0.09 [−0.25, −0.05], *p =* .05). The negative stress-reappraisal associations were significantly stronger in Japanese than in Americans, though adjustment slightly attenuated these differences (unadjusted: *z =* −3.06, *r =* −0.11 [−0.17, −0.04], *p =* .002; adjusted: *z =* −2.62, *r =* −0.09 [−0.16, −0.02], *p =* .01). Before adjustment, the inverse stress-suppression association among Japanese was stronger than the positive stress-suppression association in Americans (: *z =* −2.74, *r =* −0.09 [−0.16, −0.03], *p =* .01). However, after adjustment, these associations were stronger in Americans (*z =* −2.38, *r =* −0.08 [−0.15, −0.01], *p =* .02).

**Table 2:** Zero-order correlations for all variables stratified by culture.

		1	2	3	4	5	6	7	8	9	10	11	12	13	14	15	
Japanese	1. Stress	–	0.07	−0.19**	0.07	0.01	0.08	−0.06	0.10*	0.11*	−0.24**	0.14**	−0.05	0.06	−0.11**	0.03	Americans
	2. CVD Risk	−0.08	–	−0.03	0.07	0.71**	0.70**	0.50**	0.53**	0.62**	0.22**	−0.15**	−0.09*	0.16**	−0.23**	0.16**	
	3. Reappraise	−0.39**	0.07	–	0.26**	−0.03	−0.02	0.01	0.00	−0.04	0.02	0.02	−0.02	0.01	−0.04	0.00	
	4. Suppress	−0.12*	0.06	0.56**	–	0.10*	−0.01	0.03	0.11*	0.03	−0.02	−0.19**	0.04	0.07	−0.10*	−0.01	
	5. Log IL6	−0.01	0.60**	−0.01	0.03	–	0.58**	0.35**	0.15**	0.42**	0.41**	−0.09*	−0.09*	0.01	−0.15**	0.26**	
	6. Log CRP	0.01	0.60**	0.10	0.04	0.50**	–	0.20**	0.21**	0.43**	0.08	0.10*	−0.09*	0.13**	−0.21**	0.18**	
	7. SBP	−0.10	0.57**	0.07	0.06	0.25**	0.15**	–	0.12**	0.21**	0.31**	−0.24**	0.00	−0.11*	−0.08	0.14**	
	8. Log T/HDL	−0.02	0.59**	0.00	0.03	0.14*	0.27**	0.20**	–	0.26**	−0.07	−0.23**	−0.14**	0.23**	−0.13**	−0.06	
	9. BMI	0.00	0.61**	−0.06	0.00	0.20**	0.32**	0.31**	0.44**	–	0.05	−0.05	0.03	0.17**	−0.16**	0.15**	
	10. Age	−0.19**	0.34**	0.12*	0.23**	0.37**	0.16**	0.42**	0.07	0.10	–	−0.13**	0.07	−0.23**	0.02	0.31**	
	11. Sex	−0.02	−0.35**	0.06	−0.01	−0.13*	−0.24**	−0.27**	−0.31**	−0.31**	−0.06	–	−0.08	0.14**	−0.02	−0.01	
	12. Smoke	−0.09	−0.09	0.08	0.00	−0.05	−0.14*	−0.01	−0.13*	−0.14*	0.16**	0.28**	–	0.11*	0.24**	0.13**	
	13. Drink	−0.02	0.06	0.00	0.05	−0.03	0.02	−0.07	0.14*	−0.01	0.06	0.33**	0.20**	–	−0.08	−0.09*	
	14. Edu	0.01	−0.11*	0.03	−0.02	−0.21**	−0.04	−0.07	0.01	−0.04	−0.22**	−0.26**	0.03	−0.13*	–	0.04	
	15. Rx	−0.06	0.14*	0.04	0.05	0.22**	0.10	0.17**	−0.09	0.08	0.38**	−0.05	0.00	0.04	−0.10	–	

Note: Table 2 shows Pearson's zero−order correlations stratified by culture between the Perceived Stress Scale (Stress; Cohen et al., 1983); composite cardiovascular risk score (CVD Risk); cognitive reappraisal and expressive suppression measured using a shortened version of the Emotion Regulation Questionnaire (Gross & John, 2003); natural log-transformed interleukin-6 in pg/mL (Log IL-6), natural log-transformed C-reactive protein in mg/L (Log CRP); systolic blood pressure in mm/Hg (SBP); natural log-transformed ratio of total to high-density lipoprotein cholesterol in mg/dL (T/HDL), body mass index calculated in kg/m2 (BMI), age, sex, smoking status (Smoke), frequency of weekly alcohol intake (Alcohol), educational attainment (Education), and prescription medication use (Rx). Correlations for Japanese appear below the diagonal and Americans above the diagonal. **p < .01. *p < .05.

**Table 3:** Partial Pearson's correlations for all variables stratified by culture.

		1	2	3	4	5	6	7	8	9	
Japanese	1. Stress	–	0.12**	−0.20**	0.09*	0.10*	0.06	0.03	0.11*	0.11*	Americans
	2. CVD Risk	−0.03	–	−0.05	0.02	0.67**	0.70**	0.46**	0.51**	0.59**	
	3. Reappraise	−0.37**	0.08	–	0.26**	−0.05	−0.04	0.00	−0.01	−0.05	
	4. Suppress	−0.08	−0.02	0.55**	–	0.09*	−0.02	−0.01	0.04	−0.01	
	5. Log IL6	0.05	0.54**	−0.03	−0.05	–	0.57**	0.25**	0.13**	0.39**	
	6. Log CRP	0.03	0.55**	0.12*	0.01	0.47**	–	0.20**	0.20**	0.39**	
	7. SBP	−0.03	0.46**	0.05	−0.04	0.07	0.04	–	0.11*	0.19**	
	8. Log T/HDL	−0.02	0.53**	0.03	0.01	0.10	0.18**	0.12*	–	0.22**	
	9. BMI	0.00	0.56**	−0.05	−0.02	0.13*	0.23**	0.23**	0.37**	–	

Table 3 shows Pearson's partial correlations stratified by culture between the Perceived Stress Scale (Stress; Cohen et al., 1983); composite cardiovascular risk score (CVD Risk); cognitive reappraisal and expressive suppression measured using a shortened version of the Emotion Regulation Questionnaire (Gross & John, 2003); natural log-transformed interleukin-6 in pg/mL (Log IL-6), natural log-transformed C-reactive protein in mg/L (Log CRP); systolic blood pressure in mm/Hg (SBP); the natural log-transformed ratio of total to high-density lipoprotein cholesterol in mg/dL (T/HDL), body mass index calculated in kg/m2 (BMI), controlling for age, sex, smoking status, frequency of weekly alcohol intake, educational attainment, and prescription medication use. Correlations for Japanese appear below the diagonal and Americans above the diagonal. **p < .01. *p < .05.

### Cultural differences in CVD risk and biological variables

Japanese showed an inverse unadjusted association between stress and CVD risk, which was attenuated after adjustment (unadjusted: *r =* −0.08 [−0.18, 0.03], *p =* .18; adjusted: *r =* −0.03 [−0.14, 0.08], *p =* .56; Table 4, Table 5). Although the unadjusted association was not statistically significant, it was similar to Roy et al.’s (*r* = 0.10). In contrast, the unadjusted stress-CVD risk association in Americans was positive and became more robust after adjustment (unadjusted: *r* = 0.07 [−0.01, 0.16], *p =* .10; adjusted: *r* = 0.18 [0.08, 0.28], *p =* .001).

**Table 4:** Linear regression of CVD risk by stress and reappraisal.

	Japanese						Americans					
	*b*	*SE*	95%CI	*t*	*p*		*b*	*SE*	95%CI	*t*	*p*	
Stress	-0.002	0.03	-0.07, 0.06	-0.05	.96		0.07	0.03	0.02, 0.13	2.51	**.01**	
Reappraisal	0.45	1.12	-1.76, 2.65	0.40	.69		0.10	0.88	-1.63, 1.84	0.12	.91	
Age	0.05	0.01	0.03, 0.07	5.05	**<.001**		0.05	0.01	0.03, 0.07	4.68	**<.001**	
Sex	-1.78	0.25	-2.27, -1.30	-7.22	**<.001**		-0.98	0.24	-1.45, -0.50	-4.06	**<.001**	
Smoke	-0.35	0.34	-1.01, 0.32	-1.03	.30		-0.86	0.35	-1.55, -0.17	-2.46	**.02**	
Alcohol	0.19	0.06	0.08, 0.30	3.29	**.001**		0.42	0.09	0.24, 0.60	4.51	**<.001**	
EduAttain	-0.18	0.07	-0.33, -0.04	-2.56	**.01**		-0.27	0.08	-0.43, -0.11	-3.33	**.001**	
Rx	-0.05	0.24	-0.52, 0.42	-0.22	.83		0.43	0.29	-0.13, 1.00	1.51	.13	
Stress x Reappraisal	-0.01	0.04	-0.08, 0.07	-0.13	.90		-0.01	0.04	-0.09, 0.06	-0.29	.78	
**Conditional Effects**												

	***b***	***SE***	***t***	***r***	**95%CI**	***p***	***b***	***SE***	***t***	***r***	**95%CI**	***p***

Low	-0.002	0.03	-0.05	-.01	-0.22, 0.21	.96	0.05	0.03	1.90	.13	-0.01, 0.27	.06
High	-0.01	0.02	-0.29	-.02	-0.15, 0.11	.77	0.04	0.02	1.95	.11	0.00, 0.22	.05

**Slope Differences (Japanese v. Americans)**				
	***z***	***r***	**95% CI**	***p***
Low	-1.07	-.06	-0.18, 0.05	.29
High	-1.47	-.06	-0.14, 0.02	.14

*Note:*Table 4 shows the regression of the interaction between the Perceived Stress Scale (Stress; Cohen et al., 1983) and median split cognitive reappraisal (Stress x Reappraisal) measured using a shortened version of the Emotion Regulation Questionnaire (Gross & John, 2003) on the composite cardiovascular risk score. Unstandardized regression coefficients (b) and their associated standard errors (SE), t–values (t), effect size (r), and associated 95% confidence intervals (95%CI) for the conditional effects of the median-split reappraisal groups (low and high) on the composite cardiovascular risk score are presented separately for Japanese and Americans. Slope differences test the difference between Japanese and Americans’ low and high reappraisal slopes. Z-statistics (z), p-values, effect sizes (r), and associated 95% confidence intervals (CI) are reported. Japanese are from the Midlife in Japan study, and Americans are from the Midlife in the United States study.

**Table 5:** Linear regression of CVD risk by stress and suppression.

	Japanese						Americans					
	*b*	*SE*	95%CI	*t*	*p*		*b*	*SE*	95%CI	*t*	*p*	
Stress	0.02	0.03	-0.04, 0.08	0.59	.55		0.08	0.03	0.03, 0.14	2.92	**.004**	
Suppression	1.08	1.02	-0.92, 3.08	1.06	.29		0.61	0.86	-1.09, 2.31	0.70	.48	
Age	0.05	0.01	0.03, 0.07	5.02	**<.001**		0.05	0.01	0.03, 0.07	4.64	**<.001**	
Sex	-1.77	0.25	-2.26, -1.29	-7.20	**<.001**		-0.97	0.25	-1.46, -0.49	-3.92	**<.001**	
Smoke	-0.35	0.34	-1.01, 0.31	-1.04	.30		-0.84	0.35	-1.53, -0.15	-2.40	.02	
Alcohol	0.18	0.06	0.07, 0.30	3.15	**.002**		0.41	0.09	0.23, 0.60	4.41	**<.001**	
EduAttain	-0.18	0.07	-0.32, -0.04	-2.47	**.01**		-0.28	0.08	-0.44, -0.11	-3.36	**.001**	
Rx	-0.10	0.24	-0.57, 0.37	-0.41	.68		0.43	0.29	-0.13, 1.00	1.52	.13	
Stress x Suppression	-0.05	0.04	-0.12, 0.03	-1.25	.21		-0.03	0.04	-0.10, 0.05	-0.71	.48	

**Conditional Effects**	**Japanese**						**Americans**					
	***b***	***SE***	***t***	***r***	**95%CI**	***p***	***b***	***SE***	***t***	***r***	**95%CI**	***p***

Low	0.02	0.03	0.59	.05	-0.12, 0.23	.55	0.05	0.02	2.15	.14	0.01, 0.27	**.03**
High	-0.03	0.02	-1.26	-.09	-0.23, 0.05	.21	0.04	0.02	1.82	.11	-0.01, 0.22	.07

**Slope Differences (Japanese v. Americans)**				
	***z***	***r***	**95% CI**	***p***
Low	-0.78	-.04	-0.14, 0.07	.44
High	-2.11	-.10	-0.19, -0.01	.03

*Note:*Table 5 shows the regression of the interaction between the Perceived Stress Scale (Stress; Cohen et al., 1983) and median split expressive suppression (Stress x Suppression) measured using a shortened version of the Emotion Regulation Questionnaire (Gross & John, 2003) on the composite cardiovascular risk score. Unstandardized regression coefficients (b) and their associated standard errors (SE), t–values (t), effect size (r), and associated 95% confidence intervals (95%CI) for the conditional effects of the median-split suppression groups (low and high) on the composite cardiovascular risk score are presented separately for Japanese and Americans. Slope differences test the difference between Japanese and Americans’ low- and high-suppression slopes. Z-statistics (z), p-values, effect sizes (r), and associated 95% confidence intervals (CI) are reported. Japanese are from the Midlife in Japan, and Americans are from the Midlife in the United States studies.

The biomarker most strongly linked with CVD risk was BMI for Japanese (*r* = 0.61 [0.46, 0.62], *p = < 0*.001), and for Americans, it was IL-6 (*r* = 0.71 [0.55, 0.66]; see Table 4). This pattern was consistent even after adjustment (BMI-CVD: *r* = .56 [0.42, 0.59]; IL-6-CVD: *r* = .670 [0.534, 0.645]). Japanese showed a marginal negative stress-SBP association which was attenuated after adjustment (unadjusted: *r* = 0.10 [−0.20, 0.01], *p =* .08; adjusted: *r =* −0.03 [−0.14, 0.08], *p =* .57). However, none of the other biomarkers were significantly associated with stress before (each *r <* 0.02, *p >* .75) or after adjustment (each *r 〈* 0.03, *p 〉* .34). In contrast, Americans showed significant positive stress-T/HDL (*r* = 0.10 [0.01, 0.18], *p =* .03) and stress-BMI (*r* = 0.11 [0.03, 0.19], *p =* .01) associations, and a marginal positive stress-CRP association (*r* = 0.08 [0.00, 0.17], *p =* .05). Only the stress-CRP association was attenuated after adjustment (*r* = 0.06 [−0.03, 0.14], *p =* .19). Interestingly, adjustment revealed a significant positive stress-IL-6 association (*r* = 0.10 [0.01, 0.18], *p =* .03).

The unadjusted and adjusted stress-CVD risk associations were significantly stronger in Americans than in Japanese (each *z* > −2.06, *r >* −0.07, *p <* .04), such that it was around four times stronger among Americans compared to Japanese. After adjustment, the stress-T/HDL association was marginally stronger among Americans (*z =* −1.84, *r =* −0.07 [−0.06, −0.13], *p =* .07).

We also found significant cultural differences in the unadjusted CVD risk associations with IL-6 and CRP (IL-6: *z =* −2.68, *r =* −0.09 [−0.16, −0.02], *p =* .01; CRP: *z =* −2.30, *r =* −0.08 [−0.15, −0.01], *p =* .02), which became more pronounced after adjustment (IL-6: *z =* −2.89, *r =* −0.10 [−0.17, −0.03], *p =* .004; CRP: *z =* −3.36, *r =* −0.12 [−0.18, −0.05], *p =* .001). Moreover, the IL-6-CVD relationship among Americans was significantly more robust than the BMI-CVD link among Japanese before and after adjustment (each *z* > −2.44, *r > .*−0.08, *p <* .02). Importantly, in Americans, the adjusted CVD risk associations with IL-6 and CRP were 1.24 and 1.26 times greater, respectively, than in Japanese.

### Median split moderation analyses

Median reappraisal and suppression scores were 10 and 9 in Japanese and 10 and 8 in Americans, respectively. Participants were categorized as having low scores if their scores fell below the median and high scores if they included and exceeded the median (Roy et al., 2018)(e.g., low: < 4.67 and high: ≥ 4.67).

**Fig. 1:**
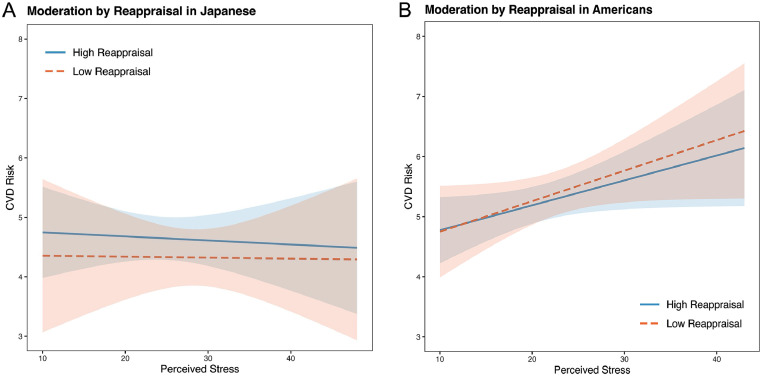
A and 1B. moderation by reappraisal in Japanese (1A) and Americans (1B).

Note: These figures show the conditional effects of low and high reappraisal on the relationship between Perceived Stress and a composite index of cardiovascular disease risk (CVD risk) among Japanese (Fig. 1A) and Americans (Fig. 1B).

**Fig. 2:**
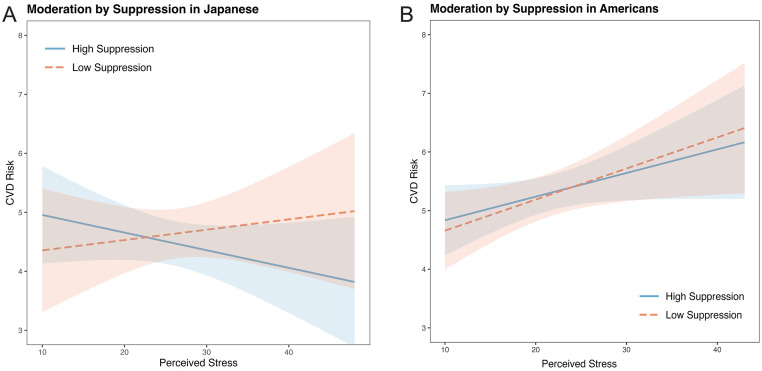
A and 2B. moderation by suppression in Japanese (2A) and Americans (2B).

Note: These figures show the conditional effects of low and high suppression on the relationship between Perceived Stress and a composite index of cardiovascular disease risk (CVD risk) among Japanese (Fig. 2A) and Americans (Fig. 2B).

Pre-planned simple slope analyses showed that among Japanese, there was no moderating effect of low or high reappraisal on the stress and CVD risk relationship (each *r* < 0.02, *p* > .77; Fig. 1A). However, among Japanese in the high suppression group, the effect of suppression on the stress and CVD risk association was negative and non-trivial (*r* = −0.09 [−0.23, 0.05]; Fig. 2A), which is similar to a marginal trend in p-values with effect sizes that are similar to or exceed those found in prior reports (Steiger, 2004). Namely, our effects exceeded Roy et al.’s (2018; High ER: *r* = 0.03 [−0.17, 0.22]). This conditional effect of higher suppression use on lower CVD risk among Japanese was over three times larger than that of higher reappraisal (*r* = −0.02 [−0.15, 0.11]). Moreover, the difference between the positive Japanese low suppression slope and the negative high suppression slope was also non-trivial (*z* = 1.24, *r* = 0.07 [−0.04, 0.18]), again exceeding Roy et al.’s (2018) effect. In contrast, among Americans, higher stress was associated with relatively higher CVD risk scores regardless of the level of suppression or reappraisal (each *r* > 0.11, *p* < .07; Figs. 1B and 2B).

The most robust cultural differences were among individuals with high suppression (*r* = −0.10 [−0.19, −0.01]; Table 5, Figs. 2A and 2B). However, consistent with Roy et al. (*z* = 0.17, *r* = 0.06 [−0.08, 0.19], *p* = .79), there were no significant differences between the low and high reappraisal slopes among Americans (each *z* < −0.82, *r* < −0.04, *p* > .41) or Japanese (each z 〈 −0.20, *r* = 0.01, p 〉 .78).

In analyses using the CVD risk score computed with only the inflammatory biomarkers (i.e., IL-6 and CRP), higher stress was associated with greater inflammation among Japanese with relatively lower suppression (*r = 0*.10 [−0.07, 0.28]) and higher reappraisal use (*r = 0*.07 [−0.05, 0.20]), and these effects were non-trivial (Steiger, 2004). In Americans, higher stress was associated with greater inflammation among those with relatively higher reappraisal use (*r* = 0.11 [0.00, 0.22]). However, no cultural differences were observed (each z 〈 −0.0.44, *r* < −0.02, p 〉 .66). Moreover, the slopes in the inflammation analyses were not significantly different relative to the slopes in the CVD risk analyses in Japanese (each z 〈 −1.09, *r* < −0.06, p 〉 .44) or Americans (each z 〈 −0.70, *r* < −0.04, p 〉 .48). See Supplemental Material. Sensitivity analyses with and without prescription medications as a covariate showed no significant differences between the slopes in each model (each r 〈 −0.04 and p 〉 .18).

## *Discussion*

Prior research has found that fewer emotion regulation difficulties attenuate the relationship between higher stress and CVD risk among Americans (Roy et al., 2018). However, comparisons of Eastern and Western populations indicate that the protective benefit of emotion regulation is differentially related to CVD risk factors as a function of culture (Miyamoto et al., 2013). The results of the present study support prior findings and indicate that even after controlling for factors that may influence CVD risk, higher suppression use may buffer the impact of stress on CVD risk among middle-aged Japanese, whereas this was not evident among Americans. Our results provide evidence of cultural differences in the relationships between stress, suppression, and CVD risk and highlight the need for future studies examining these interrelationships.

Consistent with prior reports, all biomarkers were associated with CVD risk even after adjusting for known confounds (Fox et al., 2008; Imai et al., 2021; Kinoshita et al., 2018; Lloyd-Jones et al., 2006; Turin et al., 2016). However, significant cultural differences emerged among the IL-6 and CRP associations, highlighting the importance of inflammation in the etiology of CVD disease (Abboud et al., 2012; Abboud & Singh, 2017; Singh et al., 2014). Namely, compared to Japanese, IL-6 and CRP were most strongly related to CVD risk among Americans. Moreover, consistent with prior reports, Americans had higher IL-6, CRP, and BMI than Japanese (Coe et al., 2011; Park et al., 2020). Interestingly, although there were no statistically significant cultural differences in the link between BMI and CVD risk, BMI was the risk factor most strongly linked with CVD risk for Japanese, even after adjusting for known covariates. It is noteworthy that whereas the mean BMI for Japanese in the current study was 22.98, which is just above the ideal BMI for Japanese of 22, around 41% of Japanese had low BMI (≤24) according to AHA cutoffs (Kinoshita et al., 2018; Lloyd-Jones et al., 2010). However, post-hoc analyses using Japanese BMI cutoffs were nearly identical. Therefore, using AHA cutoffs cannot explain the link between higher BMI and elevated CVD risk among Japanese. They may be more sensitive to changes in CVD risk based on BMI, such that a standard deviation change in BMI (e.g., from 22 to 25) may have significant implications for CVD risk for Japanese (Kinoshita et al., 2018). Although our findings corroborate prior reports (Chei et al., 2007; Coe et al., 2011; Fox et al., 2008; Imai et al., 2021; Kinoshita et al., 2018; Lloyd-Jones et al., 2006; Park et al., 2020), additional examinations of cultural differences in the contribution of adiposity and inflammatory biomarkers to the stress and CVD risk relationship may further explicate reports showing lower estimated lifetime risk of atherosclerotic CVD among Japanese compared to Americans (Turin et al., 2016).

Notably, to our knowledge, this is the first study to demonstrate that suppression may buffer the effect of stress on CVD risk among Japanese, whereas it was detrimental for Americans. This suggests that suppression may be an ‘adaptive’ emotion regulation strategy for Japanese with higher stress (Butler et al., 2003; Gross & Ford, 2023; Gross & John, 2003; Kashimura et al., 2024; Tyra et al., 2023). We propose that the interaction between neurobiology and culture may explain this novel finding (Kitayama & Uskul, 2011). That is, neurobiological processes are dynamically controlled via neural signals between the brain and body (Benarroch, 1993; Hiser & Koenigs, 2018; Smith et al., 2017; Thayer et al., 2021; Thayer & Lane, 2000). Our findings suggest that these neural processes and pathways may differ as a function of cultural values potentially reflecting culturally specific socialization practices (Chiao et al., 2013; Varnum, Shi, Chen, Qiu, & Han, 2014; Kitayama & Uskul, 2011, Varnum, Shi, Chen, Qiu, & Han, 2014). For instance, early parental socialization shapes emotion regulation strategies encouraged in early childhood, such as emotional restraint to foster connection in interdependent cultures, like Japan, and expressivity in independent cultures, like the U.S. (Kim & Sherman, 2007; Kitayama et al., 1997; Kitayama & Uskul, 2011; Lebra, 1976; Markus & Kitayama, 2010; Matsumoto, 1993; Rochanavibhata & Marian, 2023; Salih et al., 2023; Zahn-Waxler et al., 1996). Patterns of emotion regulation have also been linked to interdependent and independent cultural values and self-construal (Kitayama, Gideon et al., 2010; Markus & Kitayama, 1991, 2010; Oyserman et al., 2002; Triandis & Gelfand, 1998). Therefore, if ‘adaptive’ strategies help achieve goals, such as sustaining relationships (Cole et al., 1994), we theorize that suppression is a culturally congruent emotion regulation strategy for Japanese individuals. As such, repeated use of suppression in Eastern cultural contexts may reshape neural pathways related to emotion regulation, resulting in adaptive physiological responses (Chen et al., 2020; Jamieson et al., 2013; Matsumoto, 1990, 2008; Ramzan & Amjad, 2017; Soto et al., 2011; Sun & Lau, 2018; Tsai & Lu, 2018; Weiss et al., 2022). This may also explain why negative affect, which has been meta-analytically associated with higher suppression use (Boemo et al., 2022; Gross & John, 2003), predicted elevated markers of CVD risk among Americans but not Japanese (Curhan et al., 2014; Miyamoto et al., 2013; Park et al., 2020). Thus, the interaction between neurobiology and culture could explain suppression's differential role in CVD risk among Japanese and Americans (Kitayama & Uskul, 2011; Thayer & Lane, 2000). Overall, our results suggest that suppression may not be universally maladaptive, especially when stress is high (Cole et al., 1994; Kashimura et al., 2024). However, future studies should assess whether these findings hold in prospective analyses and among individuals from other Eastern populations (e.g., Chinese).

Our finding that reappraisal and suppression were not beneficial for CVD risk in Americans differs from some reports (Appleton et al., 2014) but is consistent with others (Cundiff et al., 2019). For instance, prior reports have shown beneficial cardiovascular and proinflammatory outcomes in individuals who use reappraisal (Appleton et al., 2013, 2014; Brown et al., 2022), which is inversely related to emotion regulation difficulties (Sörman et al., 2022; Zelkowitz & Cole, 2016). Moreover, in contrast to prior work, we found no buffering effect of high reappraisal on stress and CVD risk (Roy et al., 2018). The absence of cultural differences in the protective role of reappraisal on CVD risk supports the possible explanation that reappraisal may indeed be more challenging to employ and used less frequently than other regulatory strategies, particularly in the context of stress (Brans et al., 2013; Hayes et al., 1996; Suri et al., 2015; Troy et al., 2018). On the other hand, our suppression results are consistent with reports showing detrimental cardiovascular and proinflammatory outcomes in Americans (Appleton et al., 2013; Appleton & Kubzansky, 2014; Butler et al., 2003; Ellis et al., 2019; Gross & John, 2003; Lopez et al., 2020) suggesting that our discrepant reappraisal finding may only be partially attributed to our use of a different measure of emotion regulation (Roy et al., 2018). Moreover, since Roy did not report on the ethnic makeup of their sample, potential cultural differences cannot be partitioned for comparison (Roy et al., 2018). Future studies using the full ERQ scale and other measures of emotion regulation abilities are needed to clarify these discrepancies (Gross & John, 2003; Kashimura et al., 2024; Preece et al., 2018).

Taken together, our findings underscore the importance of additional cross-cultural studies to clarify the downstream effects of varied emotion regulation strategies on the relationship between stress and health outcomes in Eastern and Western populations, as our data suggests that there may not be a universally ‘adaptive’ emotion regulation strategy in response to stress, which may have different implications for CVD risk based on culture.

## *Limitations and future directions*

The present results should be considered alongside several limitations. In Western countries, individuals in lower-status occupations (e.g., unskilled workers) are at greater risk for CVD, whereas in Japan, those in higher-status occupations (managerial and professional positions) may be more at risk for coronary heart disease (Zaitsu et al., 2019). Additionally, the Japanese diet has been linked with lower levels of pro-inflammatory markers (Coe et al., 2020). Thus, future studies should consider controlling for occupation type and dietary factors. Also, our sample was unbalanced on sex, which could have influenced CVD risk scores (Ohira, Eguchi, Hayashi, Kinuta, & Imano, 2024). However, it is unlikely that sex differences influenced our results since sex was controlled for in all adjusted models. Additionally, although work-to-family spillover stress (WTFS) has been linked with triglycerides and HDL and not IL-6 or CRP in MIDUS (Hartanto et al., 2024), future studies should investigate whether these findings are consistent in MIDJA and whether WTFS is linked with other biomarkers of CVD risk (e.g., T/HDL and SBP). It is also important to note that since the internal consistency of the reappraisal subscale was relatively low, our reappraisal results should be interpreted with caution. Moreover, we did not include LDL-C in our calculation of CVD risk, despite its association with CVD risk. However, in MIDUS and MIDJA the Friedewald equation (Friedewald et al., 1972) was used to compute LDL-C, which has been shown to be inaccurate in computing LDL-C and is problematic for the detection of very low LDL-C (Martin et al., 2013; Schectman & Sasse 1993). Therefore, T/HDL was used as it has been found to be superior to LDL/HDL (Lemieux et al., 2001; Lamarche et al., 1996). However, researchers should consider including measures of small, dense LDL particles (sdLDL) in the calculation of CVD risk as it may improve its prediction power. In particular, sdLDL has been meta-analytically linked to heart disease risk (Liou et al., 2020) and has been linked with CVD-independent LDL-C in several recent large studies (Duran et al., 2020; Hoogeveen et al., 2014; Tsai et al., 2014). However, sdLDL was not included in the present study as it was unavailable. Therefore, future studies should consider direct measures of LDL-C and sdLDL. Finally, our results should be interpreted cautiously as they were cross-sectional and may not generalize to other Eastern or Western samples.

## *Conclusion*

Our findings add to our growing understanding of cultural differences in the health consequences of emotion regulation, as this is the first study to investigate cultural differences between Japanese and American adults in the moderating role of two commonly used emotion regulation strategies, suppression and reappraisal, on the association between perceived stress and CVD risk. Our results contradicted Western studies showing that reappraisal is beneficial as it did not appear to impact the link between stress and CVD risk. Notably, greater suppression use may buffer the harmful effects of stress on CVD risk for Eastern but not Western individuals, suggesting that suppression may indeed be ‘adaptive’ for individuals from Eastern cultures. Our findings may inform clinicians in tailoring behavioral interventions for patients based on their cultural background, leading to more precise and effective strategies for improving stress-coping and reducing cardiovascular risk. While the driving mechanisms of the suppression buffering effect are still being investigated, promising avenues to explain these phenomena include cultural neuroscience research and cross-cultural prospective studies using additional inflammation markers and indices of CVD risk.

## Declaration of competing interest

The authors declare the following financial interests/personal relationships which may be considered as potential competing interests: Darcianne K. Watanabe reports financial support was provided by National Science Foundation Graduate Research Fellowship Program. None If there are other authors, they declare that they have no known competing financial interests or personal relationships that could have appeared to influence the work reported in this paper.

## Appendix Supplementary materials

Image, image 1

Image, image 2

Image, image 3

Image, image 4

Image, application 5
