# Supplementary figures and images for: Emotion suppression differentially moderates the link between stress and cardiovascular disease risk in Japanese and Americans
Source: Int J Clin Health Psychol. 2025 Feb 27;25(1):100555. doi: 10.1016/j.ijchp.2025.100555 (PMC11919598; doi:10.1016/j.ijchp.2025.100555)

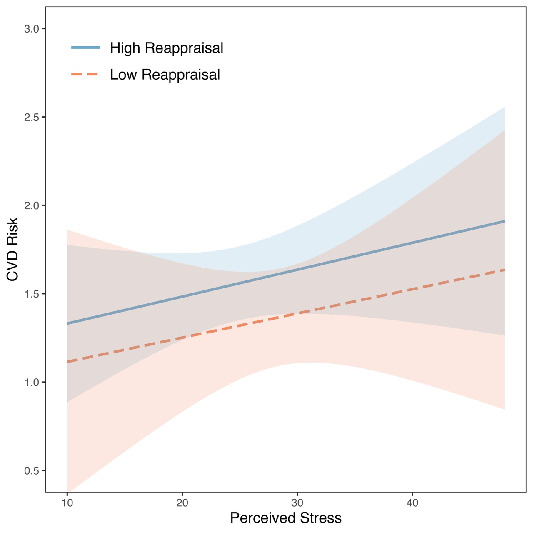

Supplement: Supplementary file 1 [file mmc1.jpg]

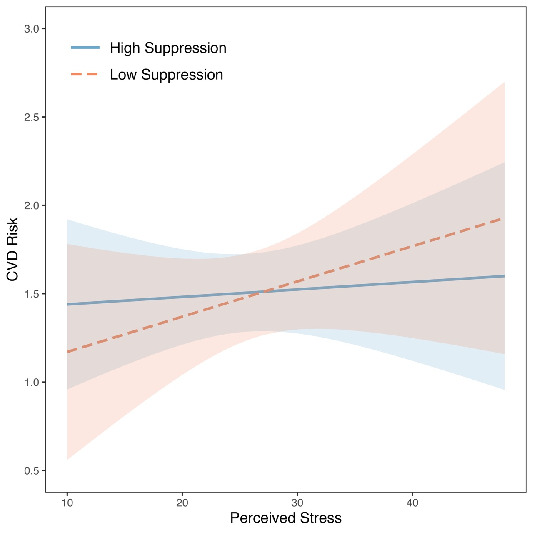

Supplement: Supplementary file 2 [file mmc2.jpg]

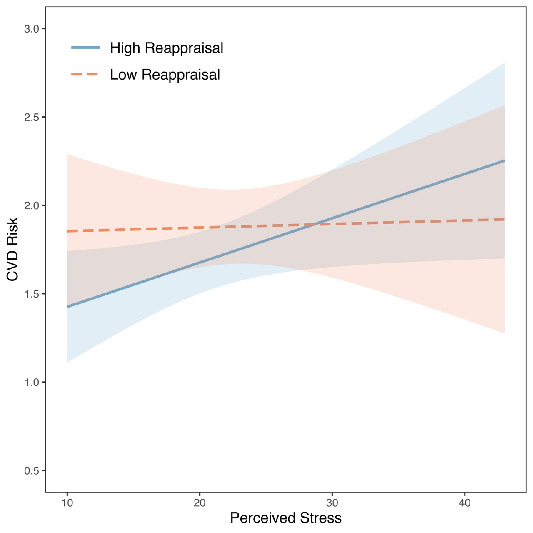

Supplement: Supplementary file 3 [file mmc3.jpg]

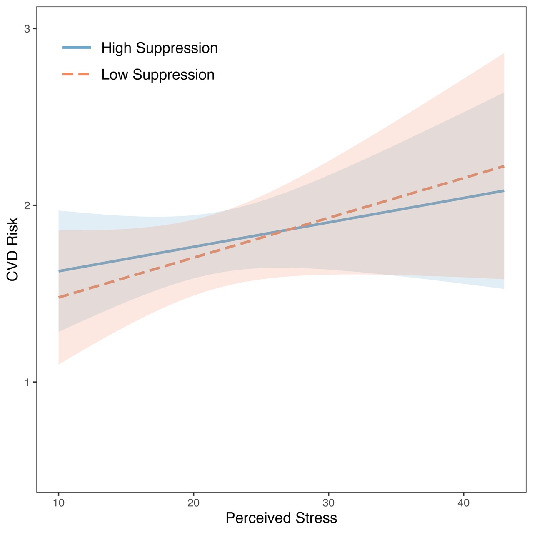

Supplement: Supplementary file 4 [file mmc4.jpg]
