# Supplementary material for: Emotion suppression differentially moderates the link between stress and cardiovascular disease risk in Japanese and Americans
Source: Int J Clin Health Psychol. 2025 Feb 27;25(1):100555. doi: 10.1016/j.ijchp.2025.100555 (PMC11919598; doi:10.1016/j.ijchp.2025.100555)
Supplement: Supplementary file 5 [file mmc5.docx]

**Table S1. Linear regression of CVD risk indexed by proinflammatory biomarkers by stress and reappraisal**

|  | **Japanese** | | | | |  | **Americans** | | | | |  |
| --- | --- | --- | --- | --- | --- | --- | --- | --- | --- | --- | --- | --- |
|  | ***b*** | ***SE*** | **95%CI** | ***t*** | ***p*** |  | ***b*** | ***SE*** | **95%CI** | ***t*** | ***p*** |  |
| Stress | -0.002 | 0.03 | -0.07, 0.06 | -0.05 | 0.96 |  | 0.00 | 0.02 | -0.03, 0.03 | 0.13 | .90 |  |
| Reappraisal | 0.45 | 1.12 | -1.76, 2.65 | 0.40 | 0.69 |  | -0.66 | 0.44 | -1.53, 0.22 | -1.48 | .14 |  |
| Age | 0.05 | 0.01 | 0.03, 0.07 | 5.05 | **<.001** |  | 0.03 | 0.00 | 0.02, 0.04 | 5.50 | **<.001** |  |
| Sex | -1.78 | 0.25 | -2.27, -1.30 | -7.22 | **<.001** |  | 0.05 | 0.12 | -0.18, 0.28 | 0.40 | .69 |  |
| Smoke | -0.35 | 0.34 | -1.01, 0.32 | -1.03 | 0.30 |  | -0.47 | 0.20 | -0.86, -0.09 | -2.41 | **.02** |  |
| Alcohol | 0.19 | 0.06 | 0.08, 0.30 | 3.29 | **.001** |  | 0.19 | 0.05 | 0.10, 0.28 | 4.14 | **<.001** |  |
| EduAttain | -0.18 | 0.07 | -0.33, -0.04 | -2.56 | **0.01** |  | -0.15 | 0.04 | -0.23, -0.07 | -3.82 | **<.001** |  |
| Rx | -0.05 | 0.24 | -0.52, 0.42 | -0.22 | 0.83 |  | 0.50 | 0.13 | 0.24, 0.77 | 3.73 | **<.001** |  |
| Stress x Reappraisal | -0.01 | 0.04 | -0.08, 0.07 | -0.13 | 0.90 |  | 0.02 | 0.02 | -0.01, 0.06 | 1.20 | .23 |  |
| **Conditional Effects** | | |  |  |  |  |  |  |  |  |  |  |
|  | ***b*** | ***SE*** | ***t*** | ***r*** | **95%CI** | ***p*** | ***b*** | ***SE*** | ***t*** | ***r*** | **95%CI** | ***p*** |
| Low | 0.01 | 0.02 | 0.71 | .08 | -0.14, 0.29 | .48 | 0.00 | 0.02 | 0.13 | .06 | -0.08, 0.20 | .90 |
| High | 0.02 | 0.01 | 1.13 | .07 | -0.05, 0.20 | .26 | 0.03 | 0.01 | 2.06 | .11 | 0.00, 0.22 | **.04** |
| **Slope Differences (Japanese v. Americans)** | | | | |  |  |  |  |  |  |  |  |
|  | ***z*** | ***r*** | **95% CI** | ***p*** |  |  |  |  |  |  |  |  |
| Low | 0.10 | .01 | -0.11, 0.12 | .92 |  |  |  |  |  |  |  |  |
| High | -0.45 | -.02 | -0.11, 0.06 | .66 |  |  |  |  |  |  |  |  |

*Note:* Table S1 shows the regression of the interaction between Perceived Stress Scale (Stress; Cohen et al., 1983) and cognitive reappraisal (Stress x Reappraisal) measured using a shortened version of the Emotion Regulation Questionnaire (Gross & John, 2003) on the composite cardiovascular risk score indexed by proinflammatory biomarkers, Il-6 and CRP (CVD Risk). Unstandardized regression coefficients (b) and their associated standard errors (SE), t–values (t), effect size (r) and associated 95% confidence intervals (95%CI) for the conditional effects of the median-split reappraisal groups (low and high) on the composite cardiovascular risk score are presented separately for Japanese and Americans. Slope differences test the difference between Japanese and Americans’ low reappraisal slopes and high reappraisal slopes. Z-statistics (z), p-values, effect sizes (r) and associated 95% confidence intervals (CI) are reported. Japanese are from the Midlife in Japan study and Americans are from the Midlife in the United States study.

**Table S2. Linear regression of CVD risk indexed by proinflammatory biomarkers by stress and suppression**

|  | **Japanese** | | | | |  | **Americans** | | | | |  |
| --- | --- | --- | --- | --- | --- | --- | --- | --- | --- | --- | --- | --- |
| Suppression | ***b*** | ***SE*** | **95%CI** | ***t*** | ***p*** |  | ***b*** | ***SE*** | **95%CI** | ***t*** | ***p*** |  |
| Stress | 0.02 | 0.02 | -0.01, 0.05 | 1.19 | .23 |  | 0.02 | 0.01 | -0.01, 0.05 | 1.59 | .11 |  |
| Suppression | 0.46 | 0.59 | -0.70, 1.63 | 0.78 | .44 |  | 0.24 | 0.42 | -0.60, 1.07 | 0.56 | .58 |  |
| Age | 0.02 | 0.01 | 0.01, 0.03 | 3.86 | **<.001** |  | 0.03 | 0.00 | 0.02, 0.04 | 5.63 | **<.001** |  |
| Sex | -0.43 | 0.14 | -0.72, -0.15 | -3.03 | **.003** |  | 0.05 | 0.12 | -0.19, 0.29 | 0.41 | .68 |  |
| Smoke | -0.31 | 0.2 | -0.69, 0.08 | -1.57 | .12 |  | -0.49 | 0.20 | -0.88, -0.11 | -2.50 | **.01** |  |
| Alcohol | 0.03 | 0.03 | -0.03, 0.10 | 0.93 | .35 |  | 0.19 | 0.05 | 0.10, 0.28 | 4.09 | **<.001** |  |
| EduAttain | -0.07 | 0.04 | -0.16, 0.01 | -1.76 | .08 |  | -0.15 | 0.04 | -0.23, -0.07 | -3.73 | **<.001** |  |
| Rx | 0.17 | 0.14 | -0.11, 0.44 | 1.21 | .23 |  | 0.50 | 0.13 | 0.23, 0.76 | 3.70 | **<.001** |  |
| Stress x Suppression | -0.02 | 0.02 | -0.06, 0.03 | -0.76 | .45 |  | -0.01 | 0.02 | -0.05, 0.03 | -0.47 | .64 |  |
| **Conditional Effects** | | |  |  |  |  |  |  |  |  |  |  |
|  | ***b*** | ***SE*** | ***t*** | ***r*** | **95%CI** | ***p*** | ***b*** | ***SE*** | ***t*** | ***r*** | **95%CI** | ***p*** |
| Low | 0.02 | 0.02 | 1.16 | .10 | -0.07, 0.28 | .25 | 0.02 | 0.01 | 1.59 | .10 | -0.02, 0.23 | .11 |
| High | 0.00 | 0.01 | 0.31 | .02 | -0.12, 0.16 | .76 | 0.01 | 0.01 | 1.08 | .06 | -0.05, 0.18 | .28 |
| **Slope Differences (Japanese v. Americans)** | | | | |  |  |  |  |  |  |  |  |
|  | ***z*** | ***r*** | **95% CI** | ***p*** |  |  |  |  |  |  |  |  |
| Low | 0.00 | .00 | -0.10, 0.10 | .99 |  |  |  |  |  |  |  |  |
| High | -0.44 | -.02 | -0.11, 0.07 | .66 |  |  |  |  |  |  |  |  |
|  |  |  |  |  |  |  |  |  |  |  |  |  |

*Note:* Table S2 shows the regression of the interaction between the Perceived Stress Scale (Stress; Cohen et al., 1983) and expressive suppression (Stress x Suppression) measured using a shortened version of the Emotion Regulation Questionnaire (Gross & John, 2003) on the composite cardiovascular risk score indexed by proinflammatory biomarkers, Il-6 and CRP (CVD Risk). Unstandardized regression coefficients (b) and their associated standard errors (SE), t–values (t), effect size (r) and associated 95% confidence intervals (95%CI) for the conditional effects of the median-split suppression groups (low and high) on the composite cardiovascular risk score are presented separately for Japanese and Americans. Slope differences test the difference between Japanese and Americans’ low suppression slopes and high suppression slopes. Z-statistics (z), p-values, effect sizes (r) and associated 95% confidence intervals (CI) are reported. Japanese are from the Midlife in Japan study and Americans are from the Midlife in the United States study.

**INSERT Figures S1A and S1B. Moderation by reappraisal on stress and CVD risk indexed by proinflammatory biomarkers**

*Note*: These figures show the conditional effects of low and high reappraisal on the relationship between perceived stress on the composite index of cardiovascular disease risk indexed by proinflammatory biomarkers, Il-6 and CRP (CVD Risk) among Japanese (Figure S1A) and Americans (Figure S1B).

**INSERT Figures S2A and S2B. Moderation by suppression on stress and CVD risk indexed by proinflammatory biomarkers**

*Note*: These figures show the conditional effects of low and high suppression on the relationship between perceived stress on the composite index of cardiovascular disease risk indexed by proinflammatory biomarkers, Il-6 and CRP (CVD Risk) among Japanese (Figure S2A) and Americans (Figure S2B).
